# Key Points from the Updated Guidelines on Exercise and Diabetes

**DOI:** 10.3389/fendo.2017.00033

**Published:** 2017-02-20

**Authors:** Sheri R. Colberg

**Affiliations:** ^1^Human Movement Sciences Department, Old Dominion University, Norfolk, VA, USA

**Keywords:** physical activity, exercise, diabetes, guidelines, American Diabetes Association

## Introduction

No doubt remains that the adoption and maintenance of physical activity is important for overall health and blood glucose management in individuals with diabetes and prediabetes. Recently, the American Diabetes Association (ADA) published updated recommendations and precautions about physical activity and exercise in people with type 1 diabetes, type 2 diabetes, and gestational diabetes (1). Given the importance of these topics, it is worth discussing the key changes and updates included in this ADA position statement (PS).

### Pre-Exercise Health Screening and Evaluation

This PS reiterates that “pre-exercise medical clearance is not necessary for asymptomatic, sedentary individuals who wish to begin low- or moderate-intensity physical activity not exceeding the demands of brisk walking or everyday living” (1). This stance directly opposes a recent recommendation from the American College of Sports Medicine (ACSM) (2) that requires anyone with a metabolic disease (in this case, diabetes) who desires to begin exercising at any level—even doing light activities—to obtain medical clearance from a health-care provider first. The authors of the ADA PS did not agree with this restriction and took the same stance as the prior ADA PS on type 2 diabetes and exercise (3), which I believe is a much better recommendation. Making adults obtain any type of medical clearance prior to starting walking, for example, is an unnecessary barrier that will not necessarily make exercising any safer for them. However, ADA agrees with ACSM that adults with diabetes who plan to exercise at higher intensities than currently undertaken or who would be considered at high risk for cardiovascular disease (e.g., have elevated blood cholesterol, smoke, have a strong family history, etc.) or other health complications from doing such activities are recommended to obtain a pre-training examination from a health-care provider who may or may not recommend exercise stress testing (3).

## Recommended Physical Activity/Exercise

All physical movement has the potential to improve physical and mental health (4–6). Since blood glucose management varies with a number of factors, it is critical for recommendations to be tailored for activity type and health complications to be effective (3, 7). In the PS, physical activity is defined as any movement that increases energy use, and exercise is a subset of physical activity that is more planned or structured (1), which is an important distinction.

### Aerobic Exercise Training

As previously recommended, most adults with type 1 or type 2 diabetes should undertake at least 150 min or more of moderate- to vigorous-intensity activity weekly; it is also recommended that these activities occur on at least 3 or more days during the week and that individuals should not allow more than 2 days to elapse between activity sessions to maintain higher levels of insulin sensitivity (Table 1). However, it is now recognized in this PS that younger or more physically fit individuals may receive similar cardiovascular and fitness benefits from undertaking vigorous-intensity or high-intensity interval training (HIIT), assuming it adds up to a minimum of 75 min/week (1, 8, 9).

**Table 1:** **Exercise training recommendations**.

	Aerobic	Resistance	Flexibility and balance
Type of exercise	Prolonged, rhythmic activities using large muscle groups (e.g., walking, cycling, and swimming)	Resistance machines, free weights, resistance bands, and/or body weight as resistance exercises	Stretching: static, dynamic, and other stretching, yoga
	May be done continuously or as high-intensity interval training		Balance (for older adults): practice standing on one leg, exercises using balance equipment, lower-body and core resistance exercises, tai chi

Intensity	Moderate to vigorous (subjectively experienced as “moderate” to “very hard”)	Moderate (e.g., 15 repetitions of an exercise that can be repeated no more than 15 times) to vigorous (e.g., 6–8 repetitions of an exercise that can be repeated no more than 6–8 times)	Stretch to the point of tightness or slight discomfort
			Balance exercises light to moderate intensity

Duration	At least 150 min/week at moderate to vigorous intensity for most adults with diabetes. For adults able to run steadily at 6 mph/9.7 kmph for 25 min, 75 min/week of vigorous activity may provide similar cardioprotective and metabolic benefits	At least 8–10 exercises with completion of 1–3 sets of 10–15 repetitions to near fatigue per set on every exercise early in training	Hold static or do dynamic stretch for 10–30 s; 2–4 repetitions of each exercise
			Balance training can be any duration

Frequency	3–7 days/week, with no more than 2 consecutive days without exercise	A minimum of 2 non-consecutive days/week, but preferably 3	Flexibility: ≥2–3 days/week
			Balance: ≥2–3 days/week

Progression	A greater emphasis should be placed on vigorous-intensity aerobic exercise if fitness is a primary goal of exercise and not contraindicated by complications; both high-intensity interval and continuous exercise training are appropriate activities for most individuals with diabetes	Beginning training intensity should be moderate, involving 10–15 repetitions per set, with increases in weight or resistance undertaken with a lower number of repetitions (8–10) only after the target number of repetitions per set can consistently be exceeded; increase in resistance can be followed by a greater number of sets and lastly by increased training frequency	Continue to work on flexibility and balance training, increasing duration and/or frequency to progress over time

*Copyright 2016© American Diabetes Association from Ref. (1). Reprinted with permission from The American Diabetes Association*.

Also included in the PS this time for the first time is HIIT, which is a type of training that includes short bursts (seconds to minutes) of very intense activity with recovery periods interspersed that may involve a lower intensity activity or rest. Such training has been demonstrated to result in greater insulin sensitivity and better overall blood glucose levels, at least in adults with type 2 diabetes (9, 10). Adults with type 1 diabetes can engage in HIIT and manage blood glucose with appropriate regimen changes (8, 11), which may include more insulin during and following and activity and reduced dosing overnight, along with food intake to prevent overnight hypoglycemia. Since its safety and efficacy remain unclear for some adults (12, 13), individuals who undertake such training should be clinically stable, already exercising regularly in activities that are moderate in intensity or harder, and possibly supervised when HIIT is started (14). This type of training is definitely not right for everyone.

### Resistance Exercise Training

The PS recommends 2–3 sessions/week of resistance exercise on non-consecutive days using a variety of strength training modalities (1), which is also unchanged from prior recommendations and from guidelines for all adults. Although heavier resistance training improves glycemic control and strength more than lighter weights or home-based activities (15), all resistance training has the potential to result in greater strength, which can translate into improved balance and ability to live independently and undertake activities of daily living.

The main PS update is related to discussing the glycemic impact of resistance exercise in adults with type 1 diabetes (1), which remains unclear (8). It may lower the risk of developing exercise-induced hypoglycemia in type 1 diabetes (16). When both aerobic and resistance exercise are undertaken during a solitary activity session, it has been shown that doing the bout of resistance work first may actually help maintain glycemic balance more so than when aerobic exercise occurs before resistance training (17). Varying the order of the activities based on blood glucose levels may minimize the risk of hypoglycemia.

### Flexibility and Balance Exercises

One major change of this PS is a greater focus on the inclusion of flexibility exercise to improve range of motion around joints in individuals of all ages (18) and balance activities to improve gait and prevent falls in older adults (19). Both flexibility exercises and balance training are recommended to be done minimally 2–3 times/week, especially by older adults (1). Including both is vitally important to living well since limited joint mobility is common in older adults and long-standing diabetes due to advanced glycation end products formed by normal aging and hyperglycemia (20). Stretching increases range of motion around joints and flexibility (18), and balance training can reduce falls risk by improving balance and gait (19).

Lower-body and core strengthening exercises may be considered part of balance training. Yoga may promote improvement in glycemic control, lipid levels, and body composition in adults with type 2 diabetes (21). Tai chi training may improve glycemic control and balance neuropathic symptoms and some dimensions of quality of life in adults with diabetes and neuropathy (22).

### Daily Movement

Engaging in more unstructured daily activity, such as errands, household tasks, dog walking, and gardening, increases daily energy expenditure and assists with weight loss and maintenance (23–25). Increasing daily movement appears to acutely lower postprandial hyperglycemia and possibly improve blood glucose management, especially when undertaken after meals (26–34). It is recommended as part of a whole-day approach and as a starting place for anyone who is currently sedentary and either unwilling or unable to start engaging in more structured activities. For many deconditioned and older individuals with diabetes, increasing daily movement may be an appropriate place to start with physical activity rather than with more structured activities.

### Reduced Sedentary Time and Interrupted Sitting

As demonstrated in adults with type 2 diabetes, encouraging them to interrupt prolonged periods of sitting with 15 min of walking after meals (26) and either light walking or simple body-weight resistance activities undertaken for 3 min after every 30 min of inactivity (29) improves overall glycemic control. The PS recommends that all adults attempt to lower the total amount of time that they spend each day in sedentary activities and break up prolonged bouts of sitting with some type of light activity for a few minutes at least every 30 min to improve their glycemic management; both should be added to daily structured exercise and unstructured movement rather than being a replacement for them. Research in this area, however, is still in its infancy—especially in populations with diabetes—and more studies are needed to better define the best types and timing of activity, not only for managing blood glucose levels but also for preventing type 2 diabetes and reversing prediabetes in the first place.

## Physical Activity and Type 2 Diabetes

The impact of exercise on insulin action is transient and, accordingly, activities should be undertaken daily or no less frequently than every other day. It is important to continue to recommend that exercise be undertaken regularly since in many cases, acute effects of aerobic exercise may not last even 24 h. At least one study has shown that if the same volume of exercise is done—either as 30 min of moderate exercise daily or 1 h at the same intensity every other day—the glycemic effects over the ensuing 48-h period are similar (35). Exercise does not necessarily need to be prolonged to result in enhanced insulin sensitivity, but if shorter in duration, engaging in harder workouts or high-intensity intervals will increase its impact (36, 37). However, daily moderate or high-intensity aerobic or resistance exercise is likely optimal (38–40). Aerobic training may improve overall glycemic control more than resistance training, but both reduce cardiovascular risk markers similarly (41), and a single bout of either may have a similar acute effect in any case (42). To achiever better glycemic management, engaging in combined aerobic and resistance training appears to be superior to undertaking either type of training on its own (43, 44). In fact, the PS states, “Adults should ideally perform both aerobic and resistance exercise training for optimal glycemic and health outcomes” (1), which I firmly believe to be an excellent recommendation.

It is also important for type 2 diabetic youth (children and adolescents) to be more physically active. Their goal should be to meet the activity goals recommended for all youth, which consists of 60 min/day or more of moderate- or vigorous-intensity aerobic activity, with vigorous, muscle-strengthening, and bone-strengthening activities at least 3 days/week (1). Few studies have been done to examine the impact of exercise training and interventions in youth with type 2 diabetes, and those are inconclusive, although it can be assumed that the health and glycemic benefits they would gain are similar to those experienced by adults with type 2 diabetes (45).

## Physical Activity and Type 1 Diabetes

This PS is the first in many decades to address the complexities of managing blood glucose with exercise in adults and children with type 1 diabetes (46). Both aerobic and resistance training are recommended for these adults (47–49), and youth with type 1 diabetes should follow general recommendations for children and adolescents (47). Blood glucose responses are impacted by the type, timing, intensity, and duration of exercise, as well as by many other factors. Different activities will likely require individualized adjustments to carbohydrate and food intake and insulin dosing during and after exercise.

Aerobic exercise after meals usually decreases blood glucose levels (50), especially during prolonged activity (34, 51, 52). Doing activity during fasting conditions, however, results in more stable glycemia, with less of a decline or even a small increase in overall levels (53). Engaging in very intense activities either maintains or raises blood glucose (16, 54), depending on duration, which is an important point to keep in mind.

Variable glycemic responses to physical activity (46) make uniform recommendations nearly impossible. In general, individuals will need to increase their carbohydrate intake and/or reduce circulating insulin levels when engaging in longer duration aerobic activities, along with frequently monitoring blood glucose. These additional recommendations are stated in the PS (1) but come from other studies: for low- to moderate-intensity aerobic activities lasting 30–60 min during fasting or basal insulin conditions, ~10–15 g of carbohydrate may suffice to prevent hypoglycemia (55). For activities done after bolus insulin, 30–60 g of carbohydrate per hour may be needed (56, 57), or insulin can be reduced 25–75% to reduce or eliminate the need for carbohydrate intake (58). Basal rate reductions for exercise may reduce hypoglycemia (59).

Continuous glucose monitors (CGM) are more widely available nowadays and have increased in accuracy; for many individuals, wearing such a device may decrease the fear of developing exercise-induced hypoglycemia. They are able to provide blood glucose trends, which can potentially assist the user in preventing hypoglycemia or treating it sooner (60–63). Some issues with CGM use during activity remain, however, as stated in the PS (1): inadequate accuracy (64), sensor filament breakage (62, 63), inability to calibrate (61) time lags between the change in blood glucose and its detection by CGM (65), and variations in sensor performance (66–68). CGM devices are currently being paired with insulin pumps into closed-loop systems run by algorithms. These technological issues with CGM use during exercise are continuing to make regular participation in physical activity a huge hurdle to creating an effective system.

## Physical Activity and Pregnancy with Diabetes

The PS recommends, “Females with pre-existing diabetes of any type should be advised to engage in regular physical activity prior to and during pregnancy” (1). It also reiterates prior recommendations from other organizations that state that “pregnant females with or at risk for gestational diabetes should engage in 20–30 min of moderate-intensity exercise on most or all days of the week” (69–71). Undertaking any type of training (aerobic or resistance) has the ability to improve insulin sensitivity and overall blood glucose management (72). Ideally, physical activity should start prior to pregnancy to reduce gestational diabetes risk (73) but can be initiated safely during pregnancy (69). Regular physical activity is important for other positive pregnancy outcomes as well and should be recommended to all females of childbearing age, both prior to and during pregnancy.

## Minimizing Exercise-Related Adverse Events

In the PS (1), it is reiterated that, “Exercise-induced hypoglycemia is common in type 1 diabetes, and to a lesser extent, people with type 2 diabetes using insulin or insulin secretagogues.” Some medications (other than insulin) may increase exercise risk, and doses may need to be adjusted (74, 75). Given that fear of hypoglycemia related to exercise is a proven barrier to exercise participation (76), any strategies that will assist in minimizing its occurrence have the potential to increase adherence to exercise training. Other acute strategies to prevent hypoglycemia involve including short sprints, performing resistance exercise before aerobic exercise in the same session, and activity timing (77–82), which are primarily based on the ability of a greater release of counterregulatory hormones during intense activities to maintain blood glucose levels more effectively. Exercise-induced nocturnal hypoglycemia is a major concern (83). Hypoglycemic events occur typically within 6–15 h postexercise (84), although risk can extend out to 48 h (85). Risk of nocturnal hypoglycemia following physical activity may be mitigated with lower basal insulin doses overnight, bedtime snacks, and/or use of CGM, and these strategies should be recommended to assist in preventing delayed-onset lows.

Very intense exercise like sprinting (79), brief but intense aerobic exercise (86), and heavy powerlifting (87, 88) may promote hyperglycemia, especially with elevated starting blood glucose levels (86). A number of strategies can mitigate exercise-induced hyperglycemia, though. For example, it may be modulated with insulin administration, interspersing moderate aerobic activity between intense bouts, and a low-intensity cooldown (89, 90). Another stance taken in the PS (1) is “Overconsumption of carbohydrates before or during exercise, along with aggressive insulin reduction, can promote hyperglycemia during any exercise (58). Exercising with hyperglycemia and elevated blood ketones is not recommended.”

Aging combined with diabetes may result in worse blood glucose control; moreover, peripheral neuropathy may be present and skin blood flow and sweating impaired (91–93), which increases the risk of heat-related illness. Chronic hyperglycemia also causes dehydration. These are all fairly new findings. For these reasons, the PS (1) recommends, “Older adults with diabetes or anyone with autonomic neuropathy, cardiovascular complications, or pulmonary disease should avoid exercising outdoors on very hot and/or humid days to prevent heat-related illnesses.”

In addition, these statements from the PS are aimed at avoidance of other exercise-related adverse responses (1), which is critical for continued participation: “Active individuals with type 1 diabetes are not at increased risk of tendon injury (94), but this may not apply to sedentary or older individuals with diabetes. Diabetes may lead to exercise-related overuse injuries due to changes in joint structures related to glycemic excursions (95), so exercise training should progress appropriately to avoid excessive aggravation to joint surfaces and structures, particularly when taking statin medications for lipid control (96).”

## Managing Health Complications

Finally, many individuals with diabetes carry the burden of having associated health concerns, many of which can impact their ability to exercise safely and effectively. None of these are new ideas, but here is a summary of recommended actions as stated in the PS (1): macrovascular and microvascular diabetes-related complications can develop and worsen with inadequate blood glucose management (97, 98). Physical activity with vascular diseases can be undertaken safely, but with appropriate precautions. Being active with peripheral neuropathy necessitates proper foot care to prevent, detect, and treat problems early to avoid ulceration and amputation. Autonomic neuropathy may complicate being active; certain precautions are warranted to prevent problems during activity, such as avoiding rapid directional changes (if orthostatic hypotension is present) and preventing dehydration and overheating during exercise with adequate fluid intake. Vigorous aerobic or resistance exercise, jumping, jarring, and head-down activities, and breath-holding should be avoided in anyone with severe non-proliferative and unstable proliferative diabetic retinopathy. Exercise with diabetic kidney disease can be undertaken safely, even during dialysis sessions. Regular stretching and appropriate progression of activities should be done to manage joint changes and diabetes-related orthopedic limitations.

## Conclusion

This PS really does not contain any controversial recommendations, other than ADA disagreeing (strongly) with the requirement that ACSM put forth that all individuals with a metabolic condition who are currently sedentary must seek medical clearance prior to getting up off the couch. It is good to be reminded as well that although everyone can benefit from being physically active, specific recommendations and precautions will vary by the type of diabetes, age, activity done, and presence of complications, and exercise prescriptions should be tailored to meet the specific needs of each individual. Overall, this ADA PS provides a comprehensive and current guide to assist individuals of all ages with any type of diabetes with engaging in recommended amounts of regular physical activity safely and effectively and is a much-needed publication. For even more specifics about type 1 diabetes and exercise participation, however, readers are referred to a very recent consensus statement (99) sponsored by the Juvenile Diabetes Research Foundation, which is far more comprehensive for this group of exercisers than this ADA PS ever intended to or realistically could be since it covered all types of diabetes, not just type 1.

## Author Contributions

SC wrote and edited this opinion piece with no input from any other authors.

## Conflict of Interest Statement

The author declares that the research was conducted in the absence of any commercial or financial relationships that could be construed as a potential conflict of interest.
